# Autism spectrum disorder: evaluation of community-based screening program

**DOI:** 10.55730/1300-0144.5822

**Published:** 2024-01-24

**Authors:** Mehmet Akif SEZEROL, Selin DAVUN

**Affiliations:** 1 Epidemiology Program, Institute of Health Sciences, İstanbul Medipol University, İstanbul, Turkiye; 2 Department of Public Health, School of Medicine, İstanbul Medipol University, İstanbul, Turkiye; 3 Sultanbeyli District Health Directorate, İstanbul, Turkiye; 4 Department of Public Health, School of Pharmacy, İstanbul Medipol University, İstanbul, Turkiye

**Keywords:** Autism, screening, autism spectrum disorder, frequency

## Abstract

**Background/aim:**

This study was conducted to evaluate the results of autism spectrum disorder (ASD) screenings conducted in a region of İstanbul between 2018 and 2023.

**Materials and methods:**

This descriptive study was conducted between April 2018 and February 2023 among 25,839 children aged between 18–36 months who had been screened for autism spectrum disorder in Sultanbeyli, İstanbul. Children between 18–36 months are examined and a form consisting of 5 questions and typical symptoms of ASD is filled. Each question is answered as yes or no. Answering yes to at least one of the questions is sufficient to direct them to child psychiatry.

**Results:**

Between 2018 and 2023, a total of 25,839 children were screened for autism spectrum disorders, 1449 children were found to be at risk, and 88 were diagnosed with autism spectrum disorder. According to the sex distribution of the children, the male:female ratio is 3.6:1. The 5-year prevalence was found to be 0.9%. With the effect of the pandemic between 2020 and 2021, screening rates have decreased and the number of diagnoses has decreased. The most common symptom among those diagnosed is delay in speaking, and the second is inability to make eye contact.

**Conclusion:**

Autism spectrum disorder is a developmental disorder whose prevalence is increasing globally and for which early diagnosis is important. To recognize this disease, it is necessary to increase screening and raise awareness among families. This study will also shed light on future studies on this subject.

## 1. Introduction

Autism spectrum disorder (ASD) is a neurodevelopmental problem that manifests in the first years of life, characterized by significant social and communicative deficits and limited, repetitive movements and interests [1]. ASD was first used by Bleuler in 1908 to describe the withdrawal observed in patients with schizophrenia.^1^ In 1943, Leo Kanner redefined the term to describe signs of social isolation and linguistic impairment in children without schizophrenia or other known psychiatric disorders. These children have difficulty communicating and interacting with others and exhibit loss of interest and repetitive behaviors in social activities [2]. Studies conducted in the field of brain anatomy, physiology, histology, and functions in the half-century that have passed since the definition of autism spectrum disorder as a disorder caused by genetic, familial, and environmental factors have revealed that this complex syndrome is a neurobiological disorder, and therefore, the individual’s social relations and communication disorders provided important data on the negative effects of their skills, interests, and behaviors [3].

Early detection of autism spectrum disorder (ASD) through developmental surveillance and screening allows children access to ASD-specific behavioral interventions that improve long-term outcomes [4].

Developmental surveillance, which includes taking anamnesis and observing for signs of ASD, lacks sensitivity, especially at short visits. In 2007, to increase sensitivity and lower the age at diagnosis of ASD, the American Academy of Pediatrics recommended screening for ASD at 18 and 24 months [5].

ASD is a neurodevelopmental disorder and special education category that has been heard frequently in our country as well as all over the world in recent years [2, 6]. In our country, the ICD-10 classification system is used for the classification of diseases. The diagnosis of ASD is made by Child and Adolescent Psychiatry Specialists in our country. In the diagnosis, the child’s development and behavior are evaluated by performing a physical examination [7]. Evaluation according to DSM-5 and ICD-10 diagnostic criteria, diagnostic tools such as Autism Diagnostic Interview–Revised (ADI-R), Diagnostic Interview for Social and Communication Disorders (DISCO), and Autism Diagnostic Observation Schedule (ADOS), Childhood Autism Rating Scale (CARS) using grading scales. Depending on the clinical situation, tests such as genetic, metabolic tests, EEG, and MRI may also be required [8].

In our country, there are a limited number of large-scale frequency studies diagnosed with ASD screening methods at the national level. In a study conducted in Türkiye, it was stated that two of the children were diagnosed with atypical autism and one with developmental delay as a result of the M-CHAT screening test applied to 2021 families with 18–36 months old children, and the prevalence of ASD in this region was reported as 1/1000 [9]. The largest research evaluating the risk of ASD in Türkiye is the autism screening project conducted by Tohum Autism Foundation in 2008. In this study, the risk of autism spectrum disorder was evaluated in approximately 45,000 children aged between 18 and 36 months. In the group screened with CHAT (Checklist for Autism in Toddlers), 15 of 228 children were reported to be at high risk, and 49 out of 242 were at moderate risk.^2^ When these data are evaluated, it is stated that there may be over 500,000 individuals with ASD and approximately 100,000 children with ASD at the age of compulsory primary education in Türkiye [10]. Within the scope of the ASD screening and follow-up program, 667,323 children were evaluated in terms of development in 2018, risk was determined in 15,087 (2.26%) of the children who underwent developmental evaluation, and 805 (0.12%) had ASD and 998 (0.15%) were diagnosed with different diagnoses (developmental delay, epilepsy, language and speech disorders, etc.). In the first three months of 2019, developmental evaluation of 220,649 children was made, risk was determined in 4921 (2.23%) of the children who underwent developmental evaluation, and 335 (0.15%) had ASD, 689 (0.31%) different diagnoses (developmental delay, epilepsy, language and speech disorders, etc.) were made.^3^

In the light of these reasons, early diagnosis of autism spectrum disorder, which maintains its importance in Türkiye and the world and continues to increase, maintains its importance and it should not be allowed to miss the diagnosis by making more frequent screenings. In particular, screening is important for primary care. This study was conducted to evaluate the children 18–36 months screened for ASD risk between 2018 and 2023 in a low socioeconomic district of İstanbul.

## 2. Materials and methods

### 2.1. Type of research

This study is a descriptive study.

### 2.2. Study population

**Figure 1:**
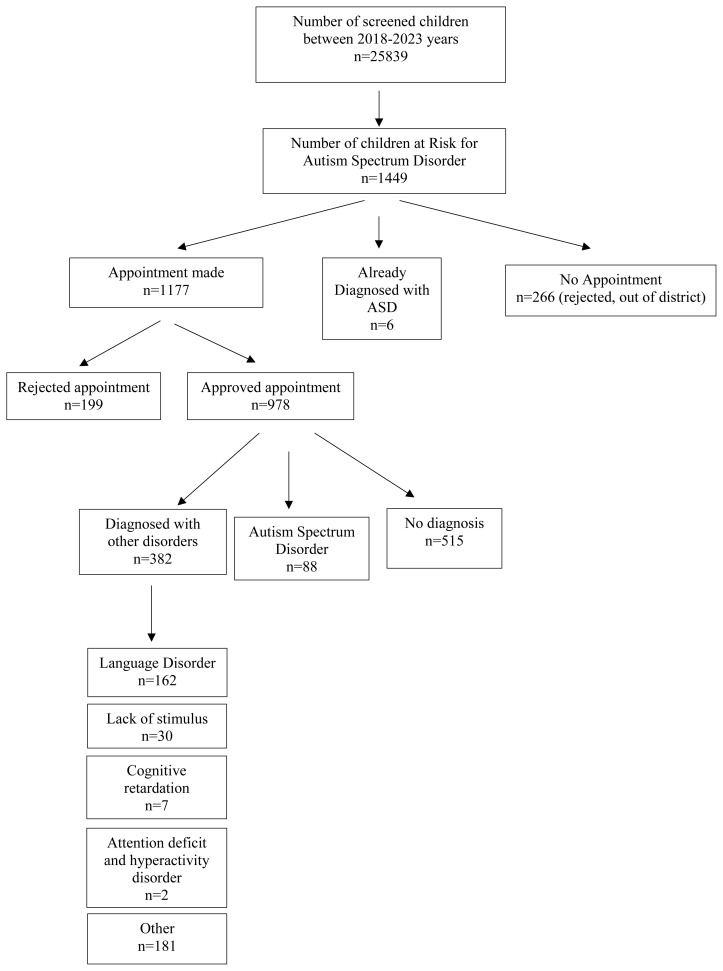
Flow chart on participants’ inclusion.

This study was conducted between 2018 and 2023 years among 1449 children aged between 18–36 months, in Sultanbeyli, İstanbul. A feature of the Sultanbeyli district is that it is the lowest district of İstanbul in the socio-economic development index. Children aged 18–36 months registered with family physicians should be screened developmentally. Since family physicians’ records are not region-based and constantly change, the target number of screenings is not known exactly. However, considering the average number of births of the last five years, it is estimated that the screening required is around 28,500. Between 2018 and 2023, 25,839 children who applied to primary care were screened and 1449 children were found to be at risk for ASD. Appointments were made for 1177 of them from child and adolescent psychiatry department. Of the 978 children who went to his appointment, 88 were diagnosed with autism spectrum disorder. Figure 1 shows the logarithm of individuals’ inclusion in the research.

### 2.3. Measuring tools

Autism screening studies in Türkiye are carried out by family physicians in primary care. In the early detection of autism determined by the Ministry of Health and applied in family physicians, it is recommended that the child between the ages of 18–36 months be evaluated according to the 5 observation items specified in the “Development Information” section of the Assessment of Psychosocial Development of the Child (CPGD) interview form [11]. The questions in the form are as follows; “Does he/she look when his/her name is called?”, “Does he/she make eye contact?”, “Does he/she look at an object you point with your finger at?”, “Does he/she have repetitive behaviors?”, “Is there a delay in his/her speech?”. Each question is answered as yes or no. Answering yes to at least one of the questions is sufficient to direct them to child and adolescent psychiatry. If at least one of them answers yes, the family doctor sends the form to the district health directorate and the district health directorate makes the child psychiatry appointment as soon as possible.

### 2.4. Statistical analysis

Descriptive data were presented with frequency tables and figures. For statistical analysis of the data, the Chi-Square test was used to compare variables. The SPSS Statistics 20.0 (Armonk, New York: IBM Corp.) statistical program trial version was used. In this study, p < 0.05 was considered statistically significant.

### 2.5. Ethical considerations

Before the study, Ethics Committee Approval and research permits were obtained from a University Ethics Committee with 871 protocol number and 13/10/2022 dated. Our study was conducted according to the Declaration of Helsinki.

## 3. Results

Between 2018 and 2023, 978 of the children aged 18**–**36 months who were screened for ASD went to a child psychiatry appointment and 88 were diagnosed with autism spectrum disorder. Three hundred and eighty-two of these children were diagnosed with other developmental disorders (language development disorder, stimulus deficiency, cognitive disorders, attention deficit, and hyperactivity disorder, etc.). While 21.6% of children diagnosed with ASD are females, 78.4% are males.

**Figure 2:**
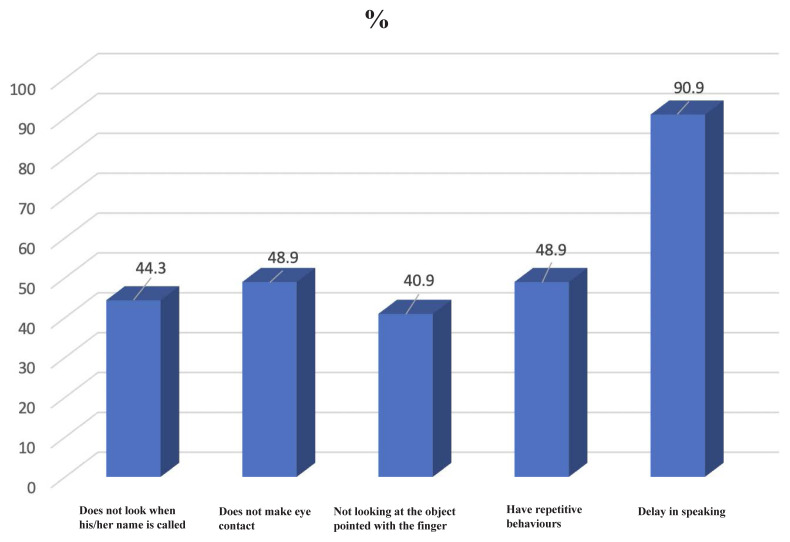
Distribution of participants according to some symptoms of ASD.

The distribution of the symptoms of the participants is shown in Figure 2. They reported the most symptoms of delay in speech with 90%.

Table shows the distribution of participants’ ASD symptoms by sex. According to the findings of this screening, not look when their name is called and having repetitive behaviors, which are among the symptoms of ASD, were more common in females, but other symptoms were more common in males.

**Figure 3:**
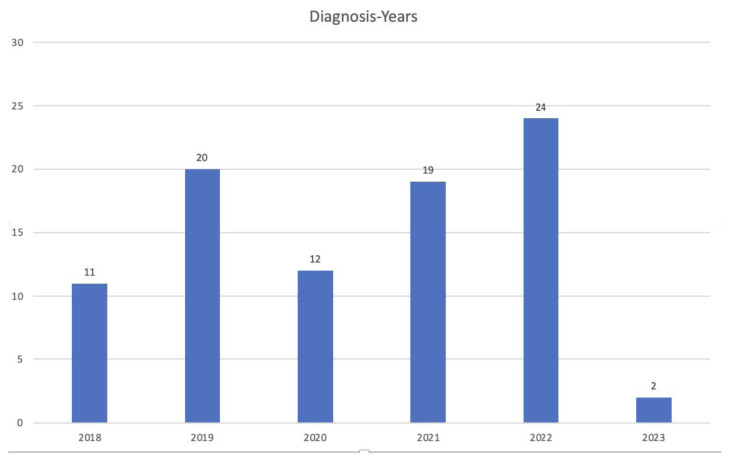
Distribution of diagnosis numbers by year.

As a result of the screening, the number of ASD diagnoses by year is as follows; 11 of children were diagnosed in 2018, 20 of children in 2019, 12 of children in 2020, 19 of children in 2021, 24 of children in 2022, and 2 of children until February 2023. The number of diagnoses by year is given in Figure 3.

The number of children screened by year and their risky status are as follows;

- In 2018, 4361 children were screened, 380 of whom were found to be at risk, and 11 were diagnosed with ASD.
- In 2019, 6382 children were screened, 267 were found to be at risk and 20 were diagnosed with ASD.
- In 2020, 3605 children were screened, 157 were found to be at risk and 12 were diagnosed with ASD.
- In 2021, 2833 children were screened, 429 were found to be at risk, and 19 were diagnosed with ASD.
- In 2022, 7186 children were screened, 429 of whom were found to be at risk and 24 children were diagnosed with ASD.
- The number of screenings until February 2023; 1472 and 60 children were found to be at risk and 2 were diagnosed with ASD. The highest number of diagnoses was made in 2022. Since the results of 2023 show only the first 2 months, it is expected to be low.

## 4. Discussion

This study was conducted with the aim of evaluating the screening results in primary care between 2018 and 2023, and a total of 1449 children were screened and 88 of them were diagnosed with autism spectrum disorder by child psychiatrists. In the region where the study was conducted, the population at risk was 9000 children on average and the prevalence of ASD was found to be 0.9%. In an ASD prevalence study conducted in Italy, the prevalence of ASD was found to be 0.3% [12]. In another study by Kim et al., the prevalence of ASD was found to be 1.9% [13]. In a review of 71 studies measuring ASD prevalence, the median value of ASD prevalence was 65/10,000[14]. Consistent with previous evidence, recent studies continue to report an increase in measured prevalence over time for certain subgroups at the country level [15, 16]. Similarly, an increase in ASD prevalence has been reported in studies of late birth cohorts in France and Australia [17, 18]. In the autism screening, Diagnosis and Development of Education Model Project in Türkiye, completed in December 2017, 9010 children aged between 18 and 36 months were evaluated in 21 districts of İstanbul and 73 of 322 children at risk were diagnosed with ASD.^5^

Considering the sex distribution of children aged 18–36 months screened and diagnosed with ASD in this study, it was found that the male sex was higher and the male:female gender ratio was 3.6. In a review evaluating the studies between the years 2000–2016 in the USA, it was stated that the ratio of male sex to female sex varies between 2.6–4.9 [19]. In another study, the male/female ratio of ASD was reported as 4.5:1 [20]. In a screening study conducted in Italy, the sex ratio of 81 children with ASD was found to be 5.2:1 [12]. Male sex is one of the best known etiological factors for ASD [21, 22]. In addition to the fact that ASD is less common in females, it is also stated that women are more likely to exhibit a more severe phenotype when they are defined as autistic. Most studies reporting IQ levels also found a higher incidence of ASD in females than males [23, 24]. However, in one study, the ratio of men with ASD without intellectual disability to women was 6–16:1, with a male/female ratio of approximately 1–2:1 for those with moderate to severe intellectual disability [25].

ASD symptoms were questioned during the screening in this study, and the most common symptom was not making eye contact after delay in speaking. In the basic clinical manifestation of ASD, stereotypical repetitive movements and restricted areas of interest are characteristic. Obsessive compulsive disorder (OCD)-like symptoms, such as arranging or collecting things in a certain order, may occur. There is atypical interest in the parts of things rather than their intended use. Persistent adherence to routines, resistance to change, and intense anxiety and restlessness in the face of change are often prominent clinical features [26]. Acquired language skills in about 1/3 of children are usually lost by age 2 [27]. Consistent with the findings of this study, language development retardation, especially in children aged 2–3 years, is an important clinical feature in terms of possible diagnosis of ASD. This screening was carried out between 2018–2023 and 2019, 2020, and 2021 coincided with the Covid-19 pandemic period, and a decrease in diagnosis rates is noticeable. The effects of the pandemic period on autism screening have been shown in studies. In a study conducted in the USA, it was determined that the screenings for ASD decreased by 8.6% during the pandemic period compared to the prepandemic period [28].

Our findings for children diagnosed with ASD also include some limitations. First of all, the 2023 findings of the study have not been completed yet. Secondly, autism screening is evaluated with 5 symptoms in primary care and does not include any scale with validity and reliability. Third, not all children aged 18–36 months, who constitute the risk group, could not be screened, which may have caused the prevalence to be low or high. Another limitation of our research is that it is estimated that there are around 28,500 children to be screened, and 25,839 children were screened. The reasons for this situation are; that not all children may have been screened, or family physicians may have performed the screening but did not enter the data into the system. Finally, out of 1449 children who were screened and found to be at risk, only 978 went to their appointment, the families of the other children refused to go or stated that they were out of the city and could not go. In addition, 200 children could not go to their appointments despite the appointment. People’s compliance with their appointments may be due to the fact that they are not yet aware of the importance of early diagnosis of the disease and that the screening is performed in a district with a low socioeconomic level.

## 5. Conclusion

Research into the epidemiology of ASD over the past decade demonstrates impressive progress in this regard. More research is needed at the community level to raise awareness of ASD. This study was conducted to evaluate the results of the screenings. Screening results provided information consistent with other studies regarding prevalence and sex distribution. The fact that people do not go to their appointments despite all the research and effort made for ASD screening also reveals the lack of awareness of the situation. Although the prevalence of ASD is gradually increasing with this screening, it also shows that screening is insufficient during the pandemic period. More research and supportive qualitative studies are needed to increase screening and raise public awareness.

## Figures and Tables

**Table:** Distribution of the symptoms according to the sex of participants.

		Sex				P-value
		Female		Male		
		n	%	n	%	
Does not look when his/her name is called	Yes	9	47.4	30	43.5	0.762
	No	10	52.6	39	56.5	
Total		19	100	69	100	
Does not make eye contact	Yes	9	47.4	34	49.3	0.883
	No	10	52.6	35	50.7	
Total		19	100	69	100	
Not looking at the object pointed with the finger	Yes	7	36.8	29	42.0	0.684
	No	12	63.2	40	58.0	
Total		19	100	69	100	
Have repetitive behaviors	Yes	11	57.9	32	46.4	0.374
	No	8	42.1	37	53.6	
Total		19	100	69	100	
Delay in speaking	Yes	16	84.2	64	92.8	0.251
	No	3	15.8	5	7.2	
Total		19	100	69	100	

## Footnotes

1 Mengi A. Sosyolojik boyutlarıyla otizm/Sociological dimensions of autism. 2014; Website: https://acikerisim.firat.edu.tr/xmlui/bitstream/handle/11508/14714/360038.pdf?sequence=1&isAllowed=y [accessed 18 August 2023]

2 Türkiye Sağlık Bakanlığı, Otizm tarama projesi sonuç raporu. 2008, İstanbul. Website: https://www.tohumotizm.org.tr/wp-content/uploads/2018/06/Saglik-Bakanligi-Otizm-Platformu-Tarama-Projesi-Raporu.pdf [accessed 5 July 2023]

3 Down sendromu, o.v.d.g., et al. 2023 Website: https://acikerisim.tbmm.gov.tr/handle/11543/3136 . [accessed 20 July 2023]

5 ohum Otizm Vakfı. Otizmde Tarama, Website:https://www.tohumotizm.org.tr/hakkimizda/projeler/tamamlananprojeler/otizmdetarama-tanilamave-egitim-modeliningelistirilmesi-projesi-2017 [accessed 29 December 2023].
